# Formation of unique nanocrystalline Cu-In-Se bulk *pn* homojunctions for opto-electronic devices

**DOI:** 10.1038/s41598-018-29457-9

**Published:** 2018-07-27

**Authors:** Shalini Menezes, Anura Samantilleke

**Affiliations:** 1 InterPhases Solar, Moorpark, USA; 2 Universidade de Minho, Braga, Portugal

## Abstract

Semiconductor *pn* junctions, integrated in optoelectronic devices require high quality crystals, made by expensive, technically difficult processes. Bulk heterojunction (BHJ) structures offer practical alternatives to circumvent the cost, flexibility and scale-up challenges of crystalline planar *pn* junctions. Fabrication methods for the current organic or inorganic BHJ structures invariably create interface mismatch and low doping issues. To overcome such issues, we devised an innovative approach, founded on novel inorganic material system that ensued from single-step electrodeposited copper-indium-selenide compounds. Surface analytical microscopies and spectroscopies reveal unusual phenomena, electro-optical properties and quantum effects. They support the formation of highly-ordered, sharp, abrupt 3-dimensional nanoscale *pn* BHJs that facilitate efficient charge carrier separation and transport, and essentially perform the same functions as crystalline planar *pn* junctions. This approach offers a low-cost processing platform to create nanocrystalline films, with the attributes necessary for efficient BHJ operation. It allows roll-to-roll processing of flexible devices in simple thin-film form factor.

## Introduction

Semiconductor *pn* junctions constitute an integral part of most optoelectronic devices, including photovoltaic (PV) solar cells and light emitting diodes (LEDs). Inorganic semiconductors offer superior carrier mobilities, light absorption/emission, and photo/thermal stability relative to organics. Conventional planar *pn* junction semiconductor devices require high quality, large mono or polycrystals. Their growth by expensive and technically complicated processes is generally incompatible with large-area processing for flexible PV and LED devices. Newer device structures, using bulk heterojunctions (BHJ) offer practical alternatives to circumvent the cost, flexibility and scale-up challenges of crystalline planar *pn* junctions. Current fabrication methods for organic, inorganic or hybrid BHJ structures use two-step process: (i) separate synthesis two types of materials followed by (ii) physical mixing, layering or dispersing nanocrystals in an organic matrix^1–11^. Such processing invariably leads to incompatible intra-device interfaces, mismatched shapes and orientations; voids and insulation due to the ligands^1,2^. Organic BHJ solar cells are limited by extremely low free carrier concentrations, low mobilities and short lifetime, although ~10% efficiency has been reported^3–7^. Inorganic BHJs use colloidal nanocrystals that are difficult to dope and thus, also constrained by extremely low free carrier concentrations^8,12^. This work introduces unique nanocrystalline material systems, and discloses a generally accessible, very low-cost method to create high quality, truly nanoscale *pn* junctions with all the necessary properties for efficient BHJ operation.

The new device and process concepts draw upon the recent R&D advancements on wide bandgap (E_g_) copper-indium-selenide (CISe) films of ordered defect chalcopyrite (ODC) compounds^13–15^. Very low ΔH creates self-stabilized CISe-ODC materials^16^. We exploited this thermodynamic advantage to devise a single-step electrodeposition (SSE) method to synthesize these materials. SSE allows controlling the film composition to produce homogeneous CISe ODC stoichiometries and is therefore, amenable for large area production^15,17^.

Besides processing advantages, the thermodynamically driven SSE process naturally creates high quality nanocrystalline CISe films with surprising nano *pn* junction attributes. SSE made nano-grained films exhibit highly desirable structural properties and mixed conductivity on a flexible metal foil. A brief air-anneal improves crystallinity, stabilizes the intrinsic defect distribution in the film and thus, enhances its photoresponse. The SSE/anneal steps can be attuned to produce specific overall film composition, comprising two types of nanocrystalline grains: *p-*CISe and *n*-CISe. Both CISe phases can absorb photons and generate free electron*-*hole pairs with high carrier density, mobility and long diffusion length. Although not appropriate for planar bilayer *pn* junction devices, this mixed-conductivity CISe film offers strong potential to function efficiently in a BHJ device configuration of: *n*-cathode/*pn*-CISe/*p*-anode. This paper highlights some salient features of SSE-made CISe films as revealed by a combination of surface analytical microscopies and spectral characterization tools.

## Results and Discussion

**Figure 1:**
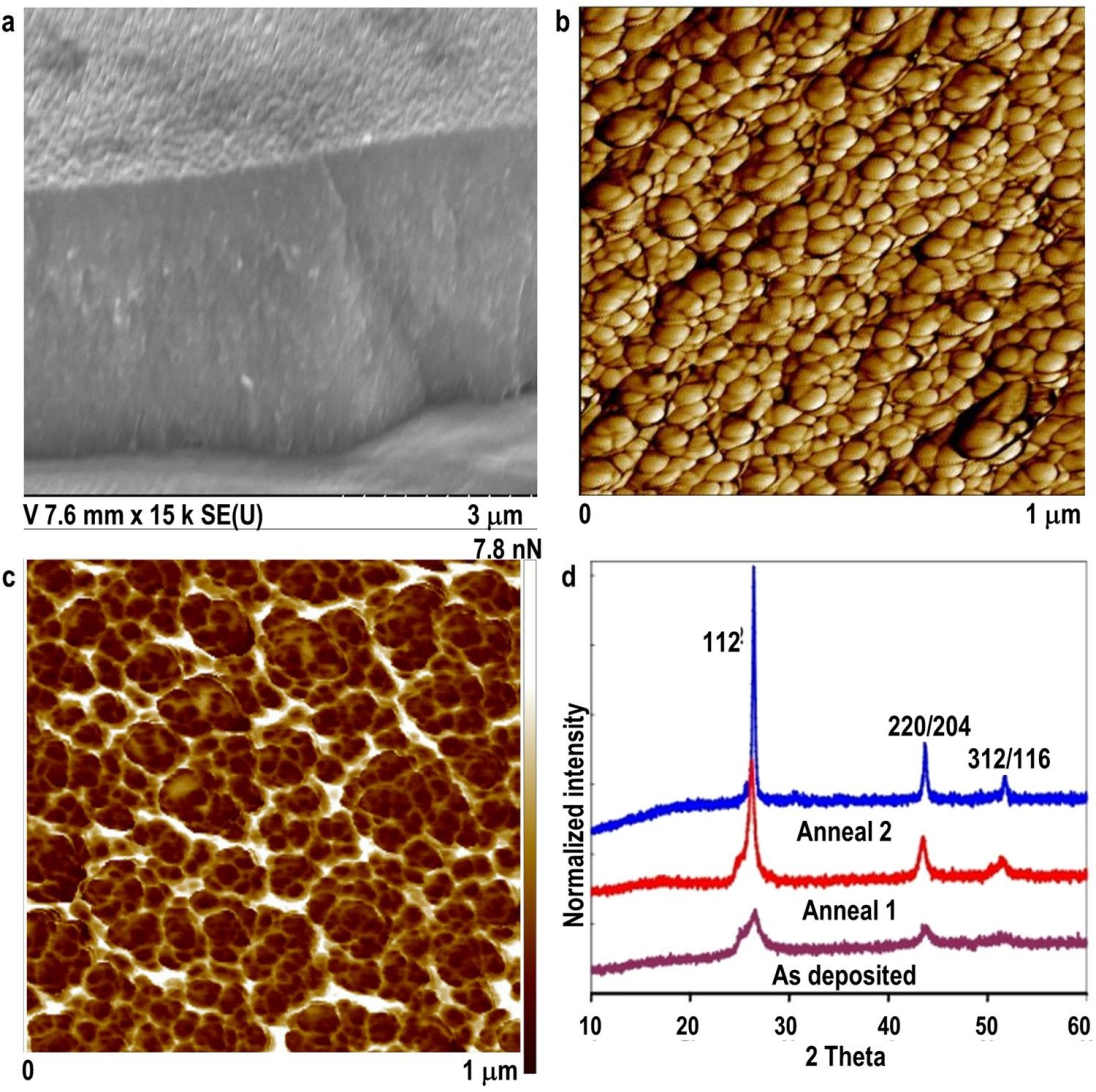
SSE-made CISe films showing (**a**) SEM, (**b**) Surface AFM, (**c**) Peak Force KPFM under 5 V AC bias, lift height: 100 nm, scan size: 1 μm, and (**d**) XRD after successive rapid thermal annealing.

The SSE/anneal combination creates smooth, shiny films with excellent topography, adhesion, and uniformity, Fig. 1: (a) scanning electron micrograph (SEM), (b) atomic force microscopy (AFM) and (c) peak force Kelvin probe frequency modulation (KPFM) adhesion force measurement. The naturally formed CISe nanocrystalline grains grow into each other; they are interlinked and space-filling, Fig. 1b. In fact, the excellent adhesion contrast at the grain boundaries indicates strong binding force (~8 nN) between the grains, which leads to a tightly packed, compact CISe film, Fig. 1c. Grain sizes can be easily controlled in the 4–35 nm range with rapid thermal processing (RTP) at relatively low temperatures, Fig. 1d.

**Figure 2:**
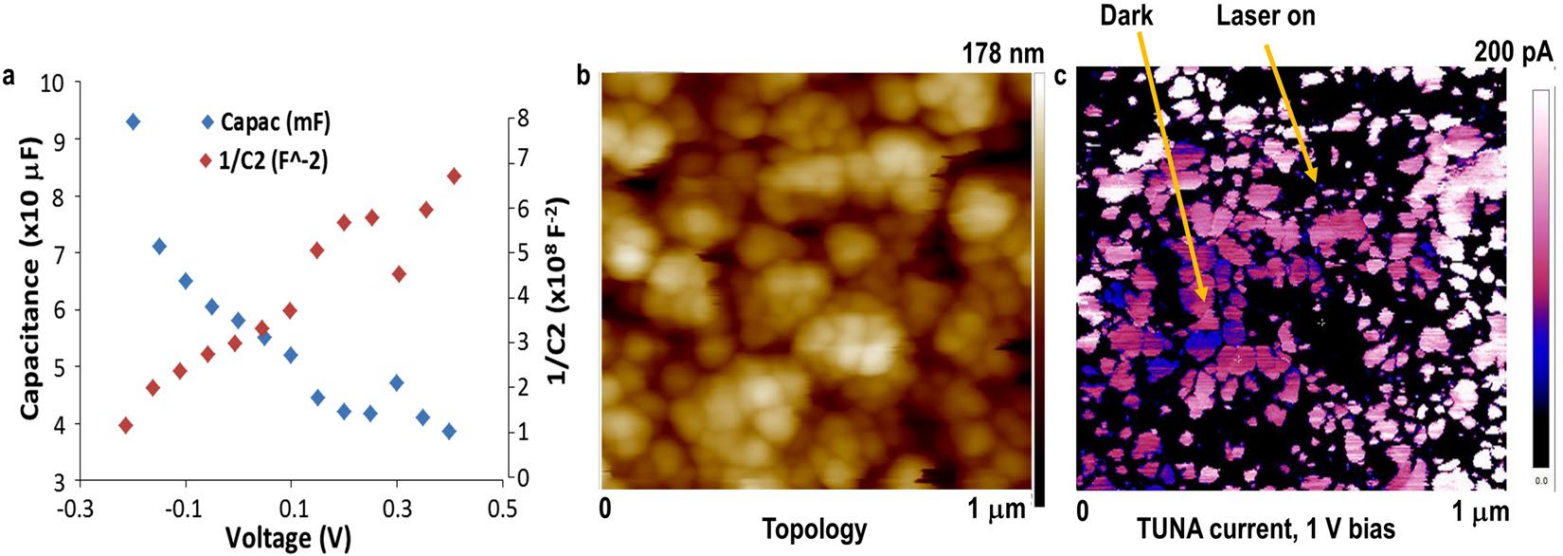
(**a**) Mott-Schottky plots of **V** *vs*. **C** and 1/**C**^2^, for CISe/SO_3_^2−^ junction (**b**) AFM topology, (**c**) dark lift tunneling current conductivity mapping under negative bias with SCM-PIT probe, DC bias: 1 V in main scan, −1 V in dark lift scan; laser on (top) and laser off (bottom section).

The Mott-Schottky plots of bias (**V**) *vs*. capacitance (**C**) and 1/**C**^2^ were obtained with electrolyte impedance spectroscopy, Fig. 2a. They indicate high capacitance, flatband potential of −0.2 V (*vs*. Ag/AgCl) and doping density (N_D_) > 10^20^/cm^3^ in the measured range. Low trapping defect density was inferred from admittance analysis of the data. It showed minimal frequency dependence of defects even when measured at 300°K. The electronic properties are similar to those for evaporated Cu(In,Ga)Se (CIGS) with higher carrier densities, lower trapping defect densities, shallower gap states and better cell performance relative to the selenized CIGS(S)^18^. The results imply that the intrinsic defects in the nano-CISe film are shallow and evenly distributed within the nano-grains and that most of these grains are uniformly and intrinsically doped (10^20^/cm^3^ ≡ one doping site per nm^3^); this can avoid degeneracy as well as contribute free carriers. The free carriers can reduce recombination, increase current and thus, enhance performance.

AFM topology and tunnelling current mapping of a CISe film provides direct evidence for the formation of adjacent *n*-CISe and *p*-CISe grains. The AFM topology illustrates the height variation of the nanocrystals, Fig. 2b. The tunnelling current mapping was obtained on the same area under negative DC bias of −1 V with the laser off (bottom) and laser on (top), Fig. 2c. Much larger current is seen under negative bias for the *n*-type grains. The surface electronic microstructure in Fig. 2c distinguishes the two types of grains distributed in the film. The high current (light area) arises from *n*-CISe grains, the low current (dark area) arises from *p*-CISe grains and the boundaries between light and dark areas evidently correspond to *pn* junctions. The CISe film is most likely completely depleted because the minority carrier diffusion length of CISe would be larger than the grain size, *i*.*e*. all the minority carriers would be swept across the nano *pn* junctions. As a result, the currents going through the *p*-grains come from hole-conduction only; this can be orders of magnitude slower than electron-conduction, because the heavier holes move slower, Fig. 2c. Comparison of the topology and tunnelling current micrographs further shows that the really prominent, bright agglomerates of nanograins seen in the topography in Fig. 2b correspond to the dark, low current spots in Fig. 2c, possibly because these agglomerates comprise depleted *pn* junctions with neutralized electrical charges. The opposite but equivalent conditions prevail for *p*-type grains under positive DC bias of 1 V, where the boundaries between light and dark areas also correspond to nano *pn* junctions in the film and these nano junctions are similarly depleted. In both cases, because there are few or no minority carriers to recombine in the *p*-grain or *n*-grain, recombination loss would be minimized for devices, such as solar cells.

**Figure 3:**
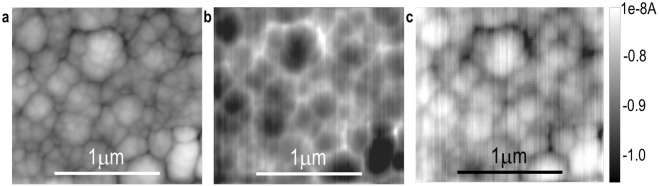
EBIC of steel/CISe/ITO device at 10 kV, showing (**a**) secondary electron topography and current mapping with the amplifier (**b**) connected to *p* side and (**c**) connected to *n* side.

Electron beam induced current (EBIC) mapping provides clear evidence for the formation of *pn* BHJ nanostructure in the CISe film. The secondary electron topography in Fig. 3a shows distinct spatial resolution of grains similar to the AFM topology in Fig. 2b. The bright EBIC contrast in Fig. 3b indicates the presence of depletion regions within the nano-scale *pn* junctions. It clearly demonstrates a 3-dimensional network of nano *pn* junctions in the CISe film. The EBIC signal is strongest in the spaces between the large grains, which most likely comprise agglomerates of smaller grains with the same type of conductivity. The opposite EBIC contrast is observed when the current is measured from the other side of the junction and the polarity of the EBIC amplifier is switched, Fig. 3c, further authenticating the data. It verifies that the EBIC actually images the *pn* junctions and that the signal response is not related to topology.

The very sharp contrast in both EBIC and AFM data indicates that the interfaces between the *p-*CISe and *n*-CISe phases are abrupt and the *pn* junctions are of very high quality. Such nano *pn* homojunctions create an electrical potential gradient, ∇*U*, at the interface between the grains. The short distance to the nano *pn* interface enables fast separation of electron and hole carriers. The continuous generation of separated electrons and holes contributes to the high carrier density; it creates and maintains a chemical potential gradient, ∇μ, which drives the transport of electrons and holes separately along the *p-*CISe and *n*-CISe phases. Note that standard bilayer planar *pn* junctions mostly utilize the ∇*U* effectively, while exitonic BHJ devices mostly utilize the ∇μ^19^. Unlike either of such device configurations, the CISe BHJ has the potential to effectively utilize both of the fundamental driving forces, i.e. ∇*U ≈* ∇*μ*.

**Figure 4:**
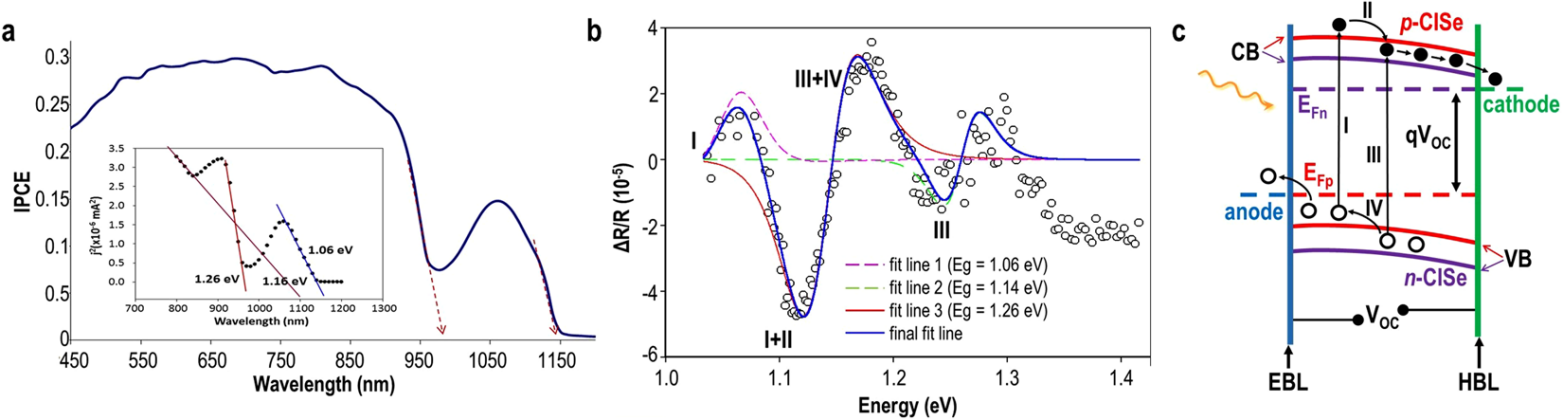
Spectral response for CISe/SO_3_^2−^ junction: (**a**) EQE spectral output and (photocurrent)^2^ *vs* wavelength (inset) at +0.5 V, showing 3 x-axis intercepts; (**b**) EER spectrum (open-circles), partial fit lines (colored dashed lines) for different parts of the spectrum and the resultant fit line (dark blue-solid line) provide bandgap & Γ values, shown in Table 1; (**c**) Band diagram, illustrating corresponding EER transitions: (I) at 1.04 eV from Γ_*p*VB_ → Γ_*p*CB_ within *p-*CISe; (II) from Γ_*p*CB_ → Γv_*n*CB_; (III) at 1.26 eV from Γ_*n*VB_ → Γ_*n*CB_ within *n-*CISe; (IV) from Γ_*n*CB_ → Γv_*p*CB_ in *p-*CISe/*n*-CISe BHJ device.

Figure 4a shows external quantum efficiency (EQE) spectrum for the CISe sample of Fig. 2a in a photoelectrochemical (PEC) cell containing 0.1 M Na_2_SO_3_ (pH 3) electrolyte. Two sharp onsets clearly indicate the presence of more than one compound. The onset values of 980 nm and 1160 nm correspond to the bandgaps of CuInSe_2_ and CuIn_3_Se_5_, Table 1. Furthermore, these two compounds appear to couple and form an energetically stable complex as indicated by a third intercept of the x-axis at 1.16 eV, shown in the spectrum analysis (inset) and in Table 1.

**Table 1:** Bandgap & Γ values from EQE and EER analysis of CISe film.

	Bandgap (eV)			Γ meV	CISe compound
	Theory	EQE	EER		
i	1.26	1.26	1.26	42	CuIn_3_Se_5_
ii	1.21*	1.16	1.14	62	CISe complex
iii	1.04	1.06	1.06	59	CuInSe_2_

*Bandgap derived for CuIn_2_Se_3.5_^16^.

Figure 4b shows electrolyte electroreflectance (EER) spectrum for the same CISe sample obtained in a PEC cell using 200 mV modulation at 13.75 Hz, superimposed on 0 V DC bias potential. The EER spectrum was very reproducible. It required three data sets for the fit^20^. The EER spectrum shows band edge of the CuIn_3_Se_5_ compound at 1.26 eV, which is an exciton-related emission. This bandgap was also associated with a very low broadening parameter, Γ of 42 meV, suggesting excellent crystal quality. The analysis revealed two more bandgaps of 1.14 eV and 1.06 eV, also with relatively low Γ values. The sharp EER peaks for electron transitions and the low Γ values indicate high degree of structural and compositional order and well crystallized chalcopyrite structure. Normally, only single crystal CISe or co-evaporated CIGS films show similar sharp peaks with very low Γ (17–30 meV) values^21,22^. In contrast, conventional electrodeposited CuInSe_2_ films do not show the three levels; their EER spectra show high Γ values (100–400 meV), indicating structural imperfections or sphalerite structure^23,24^.

The bandgaps of 1.26 eV and 1.06 eV closely correspond to theoretically derived values in Table 1. The bandgap value derived from the red fit curve of 1.14 eV is analogous to that derived from the EQE analysis of 1.16 eV and both are somewhat lower than the theoretical bandgap for CuIn_2_Se_3.5_.

The two bandgaps derived from the EER spectrum of Fig. 4b are analogous to those given by the EQE plot of Fig. 4a, verifying the presence of CuInSe_2_ and CuIn_3_Se_5_ compounds, Table 1. But the surprising features of the EER plot are the dominant middle peaks (red fit curve). The bandgap value of 1.14 eV derived from the red fit curve is close to that derived from the EQE analysis, Fig. 4a, and both values are lower than the bandgap reported for CuIn_2_Se_3.5_^16^, Table 1. These middle peaks may be attributed to the formation of a CISe complex, comprising CuInSe_2_ and CuIn_3_Se_5_ compounds. The band diagram in Fig. 4c tentatively assigns the four EER energy peaks to band-to-band electron transitions from the two CISe phases in the BHJ device. The two middle peaks corresponding to the red fit curve may each combine two transitions: (I + II) and (III + IV). Therefore, the EER spectrum in Fig. 4b is dominated by the action of *pn* junctions that leads to the separation of minority carriers across *pn* junctions (II, IV).

Because both *p-*CISe and *n-*CISe nanocrystals are absorbers, the entire BHJ structure actively contributes to current generation. A number of photocurrent measurements indicate a propensity for the CISe BHJ film to generate high photocurrent (unpublished), comparable to that for large-grained CIGS^25^. Furthermore, high open circuit potential is also attainable if the BHJ device comprises appropriately band-aligned contact electrodes, Fig. 4c. Contact electrodes with very low and very high work functions can maximize the open circuit potential. Under illumination, the Fermi level in the CISe layer splits into quasi-Fermi levels for holes (E_Fp_) and electrons (E_Fn_), creating the device open circuit potential, V_OC_. To maximize the V_OC_, the difference in the work functions of the contact electrodes should be larger than the difference between the quasi Fermi levels. Heavily doped *p*^+^ or *n*^+^ contacts can move the conduction band (CB) and valence band (VB) edges to create large band offsets. Alternately, electron blocking (EBL) & hole blocking (HBL) layers may be inserted as shown, to maximize the fill-factor.

Semiconductor behaviour is related to its fundamental properties. CISe-ODCs are unique because they form a series of stable compounds with bandgaps ranging from 1.06–1.34 eV that are intrinsically doped by shallow donor (In_Cu_, V_Se_) and acceptor (V_Cu_) defects^18,26,27^. In nanocrystalline state, the individual grains can only have either *p-* or *n*- type doping, but both types can co-exist in different grains, as is observed for CISe. Based on this understanding, we can predict that other II-VI chalcogenides could also form nanocrystalline *pn* BHJ with high doping density. This work, thus, presents a general, very low-cost ***platform technology*** to create high quality nanostructured *pn* BHJ material systems for high performance devices.

**Figure 5:**
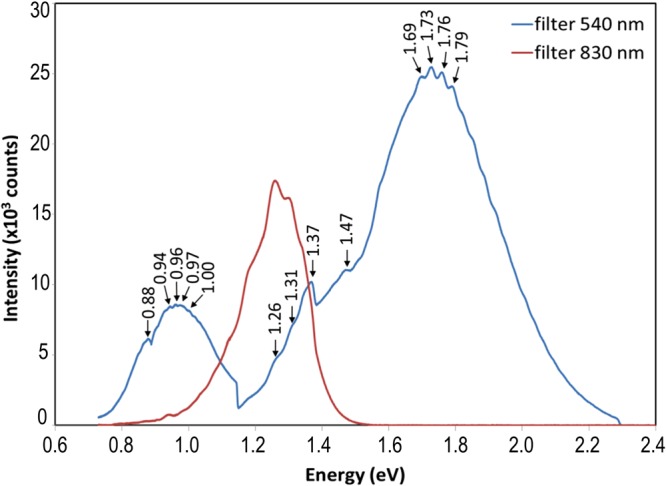
PL spectrum for CISe film, using 532 nm excitation, 830 nm and 540 nm long pass filters at 300 °K showing quantization.

Photoluminescence (PL) spectra for the CISe film in Fig. 5 are dominated by near bandgap emissions from native defects, and/or shallow donor states near the CB of CuIn_3_Se_5_ or CuIn_5_Se_8_^28^: with a 830 nm long pass filter, the PL spectrum clearly shows emissions from states near the bandgap of CuIn_3_Se_5_, such as: 1.20 eV (shallow donor state), 1.26 eV (CB), and 1.31 eV, 1.37 eV (higher energy levels inside CB, or shallow donor states from higher order CISe-ODC such as CuIn_5_Se_8_, bandgap = 1.34 eV).

With a 540 nm long pass filter, the PL response extends over a wide range of energies, 0.7–2.2 eV; it also indicates free carrier-to-shallow defect transitions, such as those from known shallow defects within the 1.06 eV bandgap of CuInSe_2_: 1.00 eV (CB →V_Cu_ or In_Cu_ →VB), 0.93 eV (In_Cu_ →V_Cu_) and 0.88 eV (V_Se_ →V_Cu_). The broadening parameters (Γ) for EER spectra in Fig. 4b also suggest similar transitions. In addition, the PL spectrum exhibits very strong, well above-bandgap emission peak, centred around 1.73 eV. This large unexpected PL peak appears to be a nanoscale phenomenon that could be attributed to alternate quantum phenomena such as: quantum confined effects in the nanocrystals that could introduce quantized energy levels into the CB; an up-conversion mechanism within the CISe *pn* BHJ nanostructures^29^; or above bandgap luminesce from accumulated high densities of electrons in quantum confined regions^30^. The spatially separated electrons and holes should exhibit long carrier lifetimes after the laser light absorption. Indeed, this specific CISe film was found to exhibit unusually long charge carrier lifetime. Substantially unequal electron paths in *n*-CISe within the CISe layer could lead to accumulation of high densities of electrons in some regions. The laser could pump and up-convert the electrons to energies well above bandgap, moving them to a different location along the *n*-CISe path. The electrons can then recombine with holes radiatively to luminesce above the bandgap. The quantum mechanical interactions of high density electrons and laser are expected to be quantized in energy and probably create the multiplet splitting with the discrete 30 mV spacing, displayed in the PL spectrum. In a solar cell, low energy (below bandgap) photons can also pump the accumulated electrons into above bandgap energy, which are essentially up-converted. The resulting broadening of the photon spectral range can significantly improve device efficiency. It is unlikely that the large PL emission peak would originate from the presence of a highly Cu-deficient phase since there is no other manifestation of this phase nor does it explain the dramatic high energy PL emission.

Both the EER and PL results could be attributed to energy transitions from crystal-field splitting and the spin-orbit coupling. Such phenomena are generally observed for large crystals under cryogenic conditions. The fact that the spectra are well-resolved even at 300°K is remarkable, and attests to the high and unique quality of our SSE-made nanocrystals. Usually, very pure and high-quality crystals or artificially created quantum well structures show such band-to-band transitions and non-linear absorption, and that too under cryogenic conditions. Moreover, the electronic nanostructure of *pn*-CISe BHJ can tolerate very wide composition variation, i.e. various CISe film compositions essentially give the same PL spectra of Fig. 5. This is in stark contrast to the normally stringent requirements for composition uniformity and material quality for processing various electronic devices and their associated high costs.

## Interpretation and Conclusions

The correlation of the surface microscopy and spectral data provide valuable insights into the extraordinary characteristics of SSE-made CISe film. Both EER and EQE spectra affirm the presence of CuInSe_2_ and CuIn_3_Se_5_ compounds and a third phase, e.g. (CuInSe_2_ + CuIn_3_Se_5_). The two types of grains arise from the specific SSE process parameter. The CISe film grows in steps that are controlled by the kinetics of several chemical and electrochemical reactions, leading to successive formation of CuSe → CuInSe_2_ → CuIn_3_Se_5_ compounds^17,31,32^. So, depending on the overall film composition, the components and defects organize into two or more compounds, each with distinct ODC stoichiometry. The EER data confirms that the SSE/anneal steps create naturally ordered CISe nanocrystals. AFM and EBIC offer unmistakable evidence that the film comprises CISe nanostructures of mixed *p*-CISe and *n*-CISe grains. The grain interface forms *pn* homojunction and possibly a stable CuInSe_2_ + CuIn_3_Se_5_ molecular complex.

The results thus imply that the CISe film comprises randomly mixed *p*- or *n*-doped nanocrystals; the random mixing of the *p*-CISe and *n-*CISe grains leads to the formation of interpenetrated and interconnected *p*- and *n-*network. The similar morphology and electronic nanostructure of the CISe-ODC compounds ensures that such a network will form, even when the *p*:*n* ratio is off by a wide margin and the individual grains have irregular shapes. The annealing step ensures that the grains grow into each other until the space between them is filled. So the grains have highly desirable matching interfaces that enable efficient BHJ operation. Significantly, this finding solves a major hurdle for creating inorganic *pn* BHJs. It avoids the need to separately synthesize nanocrystals and/or physically mix two types of materials to create BHJs, as in colloidal nanocrystals.

In general, nanocrystals tend to be perfect and are difficult to dope by extrinsic impurities^12^. Nanocrystalline CISe films are very different from colloidal nanocrystals because they exhibit high doping densities and tend to form *pn* junctions. This unique attribute of CISe nanocrystals contributes to its ordered nanoscale morphology. It avoids phase-separated domains formed in conventional BHJs, which tend to impede the interconnections needed for efficient transport of free carriers. The data supports the formation of nano-architectured *pn* BHJs that are highly ordered, sharp and abrupt. They enable fast, efficient spatial separation and transport of electrons and holes. Such processes would minimize recombination losses in solar cells, greatly relaxing the stringent requirement for large crystals in devices. In other words, the high quality 3-D *pn* BHJ can perform the same solar cell functions as 2-D planar *pn* junction, equally well.

The dramatic shift in PL emission to high energies observed for CISe films may offer an alternative type of up-conversion mechanism^30,31^. This outcome could have strong implications for accessing a wider spectral range to maximize device efficiency. Also, since the PL emissions cover most of the visible range, the nanostructured CISe film can also function as a good LED. Note that the ‘universal’ *n*-cathode/*pn-*CISe/*p*-anode device structure may be used either in solar cells or LEDs. LED structure is essentially the inverse of a solar cell. The 3D nanostructured *pn* BHJ layer is isotropic at the µm scale and current can flow through it from either direction. Thus, similar manufacturing processes can fabricate LEDs or solar cells. In theory, solar cell and LED have the same requirements for material quality and properties.

The above results validate a valuable advance in semiconductor processing. They disclose a generally accessible, very low-cost SSE method to create high quality, nanocrystalline *pn* BHJ material systems. Contrary to common perception about electrodeposited films, well-crystalized CISe nanocrystals could be used directly in solar cells or LEDs if the device is appropriately configured to take advantage of their special properties. With the incorporation of finely band-aligned contact electrode materials, the CISe BHJ film can be transformed into high performance flexible devices. The method is amenable to atmospheric, solution-based roll-to-roll manufacturing of solar cells or LEDs in a thin-film form factor. The new phenomena revealed in this work offer significant opportunity for fundamental breakthroughs in basic materials science, as well as novel applications.

## Methods

Thin film CISe samples were prepared by SSE in a standard single compartment 3-electrode cell, comprising an Ag/AgCl (+0.222 vs NHE) reference electrode (RE) and Pt or graphite counter electrode (CE). The films were deposited from a single electrolyte by varying deposition parameters to control the composition of the CISe film to produce CuInSe_2_ and CuIn_3_Se_5_ compounds in roughly 1:1 ratio. The electrolyte comprised Cu^2+^, In^3+^ and Se^4+^ ions in 0.1 M KCl. The [Cu]:[In]:[Se] metals concentration was generally maintained at 1:4:2 ratio and the solution temperature was between 50–60 °C. Most films were deposited at constant potential between −0.51± 0.01 V, using Pine Instruments potentiostat on ~5 cm^2^ exposed area of back-insulated Mo or stainless-steel foil. X-ray fluorescence analysis (XRF) with Spectro Midex provided the film composition and thickness. The films were briefly annealed in the 200–350 °C range in ambient atmosphere, using a combination of conventional furnace and RTP. Furnace annealing was done at 350 °C for up to 20 min and RTP was done with an IR lamp (650 W) at 90% with 2–3 sec pulses, repeated 10–15 times. The thermal processing requirements vary for different substrates, (glass or metal foil)^17^. The annealing was minimized in order to retain the structural order and integrity generated by the SSE process.

Tunnelling AFM measurements used bias polarity effects to identify *p*-type and *n*-type grains in the film. The AFM topological and nanoelectrical micrographs were obtained with Bruker Dimensional Icon and SCM-PIT probe on a 1 µm area of CISe sample. The dark lift mode was used to eliminate the laser effects because the electron-hole pairs generated in the *pn* junction device with laser illumination can significantly affect the minority carrier concentration, and hence the Fermi energy level and the width of the depletion region. KPFM measured the work function difference of tip/CISe sample. The measurements were done on 1 µm area of the CISe sample, using PFNQE-AL probe under AC bias of 5 V and lift height of 100 nm.

The EBIC maps were obtained using Ephemeron Labs quantitative EBIC imaging system designed for nano-electronic materials. The electron beam of an SEM was scanned across the foil/CISe/ITO device surface to generate electron–hole pairs; they separate by drift due to the electric field, causing current flow. A picoammeter detects electronic activity variations due to the presence of *pn* junctions.

**Figure 6:**
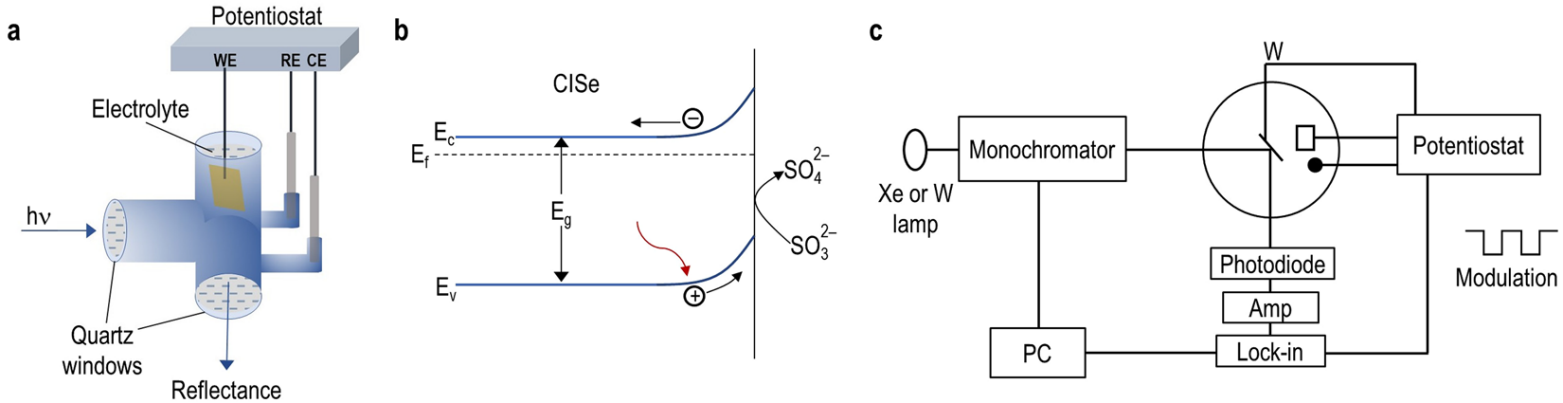
(**a**) Cell configuration used in QE and EER measurements; (**b**) Band diagram representing the electrolytic contact to CISe; (**c**) Set up for EER characterization with DC bias superimposed with an AC signal; Reflectance signal is fed to the PC controlled lock-in amplifier via a photodiode.

Electrochemical characterization was performed on masked CISe samples, exposing an area of 0.25 cm^2^ to the electrolyte held in a 3-electrode cell provided with 2 quartz windows, Fig. 6a. Na_2_SO_3_ (pH 3) electrolyte, a good hole-scavenger was used to form an electrolytic junction with the CISe sample, Fig. 6b. Figure 6c shows a schematic of the setup used for EER and EQE analysis of samples. EER spectroscopy uses an AC modulation, superimposed on a DC bias potential. This gives rise to small changes in the transmission at photon energies close to critical points in the band structure. The changes are a consequence of perturbation of the dielectric function, hence the absorption coefficient and the refractive index by the modulation. EQE spectra were measured using two different calibrated photodiodes by applying a +0.2 V bias. A Si photodiode was used between the wavelengths 400–1100 nm, and a Ge photodiode was used between 600–1200 nm, so that EQE values overlap in the intermediate wavelength range. The EER spectrum required three data sets for the fit. Still, the spectrum was very reproducible. The bandgap and Γ values in Table 1 were estimated from the EER spectral fit to the third derivate Lorentzian line function for low field modulation spectroscopies^20^. The EER responses were phase sensitively detected at room temperature temperature (non-cryogenic) and the spectrum was analysed by fitting of the experimentally obtained spectrum to the third derivative functional form. The transition energy and the broadening parameters were obtained from equation 1 as,

1 $$\frac{{\Delta }R}{R}=Re\,[{C}_{j}{e}^{i{\theta }_{j}}{(E-{E}_{gj}+{i}_{j})}^{-{m}_{j}}]$$

where Δ*R/R* is the relative reflectivity change, *E* is photon energy of the probe beam, *j* is the number of spectral function to be fitted, *Eg*_*j*_ is the critical point energy, *Γ*_*j*_, *C*_*j*_, and *θ*_*j*_ are the broadening parameter (≡ measure of disorder), amplitude, and phase angle, respectively; *m*_*j*_ corresponds to a band-to-band transition and is assumed to be 2.5.

The PL spectra were obtained using Horiba’s Micros setup with a 532 nm laser excitation at room temperature. Long pass filters were used to block the short-wave light below 540 nm or 830 nm for the spectra at respective wavelengths.
